# Are Patient Views about Antibiotics Related to Clinician Perceptions, Management and Outcome? A Multi-Country Study in Outpatients with Acute Cough

**DOI:** 10.1371/journal.pone.0076691

**Published:** 2013-10-23

**Authors:** Samuel Coenen, Nick Francis, Mark Kelly, Kerenza Hood, Jacqui Nuttall, Paul Little, Theo J. M. Verheij, Hasse Melbye, Herman Goossens, Christopher C. Butler, on behalf of the GRACE Project Group

**Affiliations:** 1 Laboratory of Medical Microbiology, Vaccine & Infectious Disease Institute (VAXINFECTIO), Faculty of Medicine and Health Sciences, University of Antwerp, Antwerp, Belgium; 2 Centre for General Practice, Department of Primary and Interdisciplinary Care Antwerp, Faculty of Medicine and Health Sciences, University of Antwerp, Antwerp, Belgium; 3 Cochrane Institutes of Primary Care and Public Health, School of Medicine, Cardiff University, Cardiff, United Kingdom; 4 South East Wales Trials Unit, School of Medicine, Cardiff University, Cardiff, United Kingdom; 5 Primary Care Medical Group, University of Southampton Medical School, Southampton, United Kingdom; 6 Julius Center for Health Sciences and Primary Care, University Medical Center Utrecht, The Netherlands; 7 General Practice Research Unit, Institute of Community Medicine, University of Tromso, Tromso, Norway; The Hospital for Sick Children and The University of Toronto, Canada

## Abstract

**Background:**

Outpatients with acute cough who expect, hope for or ask for antibiotics may be more unwell, benefit more from antibiotic treatment, and be more satisfied with care when they are prescribed antibiotics. Clinicians may not accurately identify those patients.

**Objective:**

To explore whether patient views (expecting, hoping for or asking for antibiotics) are associated with illness presentation and resolution, whether patient views are accurately perceived by clinicians, and the association of all these factors with antibiotic prescribing and patient satisfaction with care.

**Methods:**

Prospective observational study of 3402 adult patients with acute cough presenting in 14 primary care networks. Correlations and associations tested with multilevel logistic regression and McNemar ‘s tests, and Cohen’s Kappa, positive agreement (PA) and negative agreement (NA) calculated as appropriate.

**Results:**

1,213 (45.1%) patients expected, 1,093 (40.6%) hoped for, and 275 (10.2%) asked for antibiotics. Clinicians perceived 840 (31.3%) as wanting to be prescribed antibiotics (McNemar’s test, p<0.05). Their perception agreed modestly with the three patient views (Kappa’s = 0.29, 0.32 and 0.21, PA’s = 0.56, 0.56 and 0.33, NA’s = 0.72, 0.75 and 0.82, respectively). 1,464 (54.4%) patients were prescribed antibiotics. Illness presentation and resolution were similar for patients regardless their views. These associations were not modified by antibiotic treatment. Patient expectation and hope (OR:2.08, 95% CI:[1.48,2.93] and 2.48 [1.73,3.55], respectively), and clinician perception (12.18 [8.31,17.84]) were associated with antibiotic prescribing. 2,354 (92.6%) patients were satisfied. Only those hoping for antibiotics were less satisfied when antibiotics were not prescribed (0.39 [0.17,0.90]).

**Conclusion:**

Patient views about antibiotic treatment were not useful for identifying those who will benefit from antibiotics. Clinician perceptions did not match with patient views, but particularly influenced antibiotic prescribing. Patients were generally satisfied with care, but those hoping for but not prescribed antibiotics were less satisfied. Clinicians need to more effectively elicit and address patient views about antibiotics.

## Introduction

Acute cough is one of the commonest reasons for consulting and for prescribing antibiotics in primary care [1], [2]. In a study of adult patients presenting in primary care with acute cough in 13 European countries, 52.7% were prescribed antibiotics and the median time it took for patients to feel recovered was 11 days [3]. Variation in antibiotic prescribing was not associated with clinical outcome, supporting evidence from randomised controlled trials that antibiotics are over prescribed for this condition [4].

Patient expectations for antibiotics are associated with increased antibiotic prescribing, and clinicians’ belief that patients expect antibiotics are associated with even greater likelihood of prescribing [5]–[14]. Clinicians have been found to overestimate patient expectations for antibiotics, especially in patients presenting with acute cough [15], [16]. ‘Expecting antibiotics’ (patient’s perception of what the clinician might do) differs from ‘hoping for’ an antibiotic prescription [11], and from expressing hope for an antibiotic (asking for antibiotic treatment). Understanding the influence of patient expectations, hopes and actual requests for antibiotics and clinician recognition of these are important in addressing inappropriate antibiotic prescribing. However, previous research into the role of patients’ views has not taking into account the difference between expectations, hopes and asking, nor illness severity at presentation and patient recovery. This is important because those that expect, hope for and/or ask for antibiotics may be sicker or more likely to benefit from antibiotic treatment, and were this the case, then attempts to modify these expectations, hopes and requests may be inappropriate. In addition, it is unclear from current evidence whether patient satisfaction with care is associated with congruence between a clinician’s prescribing decision and patient expectations, hopes and requests for antibiotics [17], [18]. This is important because clinicians’ perceptions of how their prescribing decisions are likely to affect patient satisfaction might be a factor in non-evidence based variation in antibiotic prescribing [19], [20].

Therefore, we set out to explore 1) whether patient views (expecting, hoping for or asking for antibiotics) are associated with illness presentation or resolution, and whether these associations are influenced by antibiotic prescribing, 2) whether patient views are accurately perceived by clinicians, 3) whether patient views or clinician perceptions are associated with antibiotic prescribing, and 4) whether antibiotic prescribing influences patient satisfaction, and whether this association is influenced by patient views.

## Materials and Methods

### Study Design

This was a prospective observational study in 14 primary care networks (PCNs) in 13 European countries with clinicians recording symptoms on presentation and management. Ethics review committees in each country approved the study (Belgium (Antwerp): Medical ethics committee of the University Hospital Antwerp; Finland (Helsinki): Koordinoiva eettinen toimikunta ethics committee of Helsinki and Uusimaa Health District; Germany (Rotenburg): Ethik-Kommission der Medizinischen Fakultät Universität Göttingen; Hungary (Balatonfured): Intezeti kutatasetikai bizottsag; Italy (Milan): Segreteria scientifica del comitato etico; Norway (Tromso): Regional Komite for medisinsk forskningsetikk; Poland (Lodz): Komisja Bioetyki Uniwersytetu Medycznego W Lodzi; Solvakia (Bratislava): Etickej komisie Bratislavského samosprávneho kraja; Spain (Barcelona and Mataro): Hospital Clinic de Barcelona y precidente del CEIC (Comité Ético de Investigación Clínica); Sweden (Jonkoping): Regionala etikprövningsnämnden I Linköping; Netherlands (Utrecht): Medisch-Ethische Toetsingscommissies (METC) UMC Utrecht; UK (Cardiff (Wales) and Southampton (England)): Multi-Centre Research Ethics Committee for Wales). All participants provided their written informed consent to participate in this study. Details of the GRACE (Genomics to combat Resistance against Antibiotics in Community-acquired LRTI in Europe; www.grace-lrti.org) observational study of acute cough have been reported elsewhere [3], [21]–[26].

### Participants

Eligible patients were at least 18 years and consulting with an illness where an acute or worsened cough was the main or dominant symptom, or had a clinical presentation that suggested a lower respiratory tract infection (LRTI), with a duration prior to consulting of up to and including 28 days.

### Data

General practitioners (GPs) and nurse practitioners used a case report form (CRF) to record aspects of patients’ history, symptoms, co morbidities (diabetes, chronic lung and cardiovascular disease), clinical findings, and their management including antibiotic prescription. Clinicians indicated the presence or absence of 14 symptoms (cough, sputum production, shortness of breath, wheeze, coryza, fever during this illness, chest pain, muscle aching, headache, disturbed sleep, feeling generally unwell, interference with normal activities, confusion/disorientation and diarrhoea) and then rated whether this symptom constituted ‘no problem’, a ‘mild problem’, a ‘moderate problem’ or a ‘severe problem’ for the patient. They were also asked to record the colour of the patient’s sputum (if present). Clear or white sputum is denoted normal and yellow, green or blood-stained is denoted abnormal. They recorded the patient’s body temperature with a disposable thermometer (TempaDot, 3M Health Care), whether they had performed a lung auscultation, and if they had whether or not they found diminished vesicular breathing, wheeze, crackles, or rhonchi. The number of auscultation abnormalities detected was categorised as none, one, or two or more.

In addition, clinicians rated the extent to which they felt the patient wanted them to prescribe antibiotics and was satisfied with the consultation, on a 5-point scale (“strongly agree”, “agree”, “neither agree nor disagree”, “disagree”, or “strongly disagree”).

After the consultation, patients were given a symptom diary and asked to rate 13 symptoms each day until recovery (or for 28 days if symptoms were on-going) on a 7-point scale ranging from “normal/not affected” to “as bad as it can be”. Patients rated the same symptoms as the clinicians except for confusion/disorientation and diarrhoea. They were also asked about interference with social activities. A total symptom severity score was calculated by summing the scores for each symptom and scaling it so that it could be interpreted as a percentage symptom severity score. Patients also reported their smoking status and the day they felt recovered.

At the start of the diary, patients were asked to respond with either yes or no to the following three questions: “Were you expecting your GP or nurse to prescribe antibiotics?”, “Were you hoping that your GP or nurse would prescribe antibiotics?”, “Did you ask your GP or nurse for antibiotics?” The translations of these questions in the different languages are presented in Table S1. They were also asked to rate how satisfied they were with consultation on that day on a 5-point scale: “very satisfied”, ”satisfied”, “neither satisfied nor dissatisfied”, “dissatisfied”, and “very dissatisfied” (except in the Norwegian network where this question was not asked).

### Analysis

Descriptive statistics by network and overall were calculated by using means and standard deviations (SD), medians and interquartile range (ICR), and proportions as appropriate. Presented SDs were inflated for clustering [27]. Non-responders to the diary were compared to responders on basic demographics using t-tests, chi-square tests and Wilcoxon rank sum tests as appropriate.

#### Symptom severity and resolution

To explore whether patient expectations, hopes or asking for antibiotics are associated with symptom severity at baseline or symptom resolution over time, and whether antibiotic prescribing modifies these associations, we used a three-level hierarchical model combining an autoregressive (1) and a moving average process (1) (ARMA (1,1) model) [28], with logged daily symptom scores nested within patients nested within clinicians, and fitted three-way interaction terms between being prescribed an antibiotic, time measured in days and each of expecting, hoping for or asking for antibiotics. To illustrate the differences in symptom resolution, predicted symptom scores on days one and seven are presented for patients (not) expecting, hoping for and/or asking for antibiotics, and for each of these subgroups split by whether they were prescribed antibiotics or not.

#### Clinicians’ perception

To assess whether patient expectations, hopes or asking for antibiotics are over or underestimated by clinicians we performed McNemar’s tests [29] and to assess whether they match with clinician perceptions of patient requests we calculated Cohen’s Kappa values [30], and complemented these with values for positive agreement (PA) and negative agreement (NA) [31]. For this purpose the ratings of clinicians’ perceptions were dichotomised, grouping “strongly agree” and ”agree”, and “neither agree nor disagree”, “disagree” and “strongly disagree”.

#### Influence on antibiotic prescribing

To explore whether patient expectations, hopes or asking for antibiotics, or clinician perceptions are associated with antibiotic prescribing, we used a two-level hierarchical logistic regression model, with patients nested within clinicians.

#### Patient satisfaction

The relationship between patient satisfaction and clinicians’ perceptions was assessed in the same way as clinicians’ perceptions of patient expectations. Patients’ ratings of their satisfaction were dichotomised, grouping “very satisfied” and ”satisfied”, and “neither satisfied nor dissatisfied”, “dissatisfied” and “very dissatisfied”. To assess whether patient expectations, hopes and asking for antibiotics modify any association between antibiotic prescribing and patient satisfaction, we used a two-level hierarchical logistic regression model, with patients nested within clinician, with interaction terms between antibiotic prescribing and each of patient expectations, hopes and asking for antibiotics and clinician perceptions of both whether the patient wanted antibiotics and whether the patient was satisfied with the consultation.

All models are controlled for clinical presentation by including patient information on 13 of the 14 clinician recorded symptoms (cough was excluded as it was present in 99.8% of cases), sputum type, the number of auscultation abnormalities, temperature, age, co-morbidities (cardiovascular, respiratory, and diabetes), the duration prior to consulting, smoking status and PCN. The predicted symptom severity scores are based on an average patient, i.e. an adult with acute cough, moderately severe phlegm production, feeling moderately unwell and normal temperature (≥36°C and ≤37.2°C), median age (45), median days waited before presentation (5), no comorbidities, and non-smoker.

## Results

A total of 3,402 patients were recruited by 387 practitioners [3]. Six networks included 270 patients or more, and all included over 100. Four patients were later found to be ineligible and were therefore excluded from further analysis. CRFs were completed for 3,368 (99%) and diary data was obtained from 2,714 (80%) patients. We analysed data from 2,690 patients with both useable CRF and patient diary data (Tables 1 and S2). They were older and more often prescribed antibiotics than those not included in the analysis. There were also statistically significant differences between those included and those not in temperature, total clinician recorded symptom severity scores, proportion with a cardiovascular or diabetic comorbidity, but either the numbers were small or the differences were marginal. Otherwise their characteristics were similar to those not included in the analyses (Table S3). Their median symptom severity score was 28.6. Overall, 54.4% (1,464) were prescribed an antibiotic, but with striking variation between the PCNs [3]. The median time for patients’ symptom severity scores to drop to 0 was 12 days.

**Table 1:** Characteristics of adult patients presenting in primary care with acute cough/LRTI by primary care network (country).

		Patients							Clinician perceptions of patient				
					Satisfied with consultation, % (n)				Wanting antibiotics,% (n)		Satisfaction,% (n)		
	Patients/practises/clinicians,n/n/n	Expectingantibiotics,% (n)	Hoping for antibiotics,% (n)	Asking forantibiotics,% (n)	Very satisfiedor satisfied	Not	Symptomseverity score†,median(ICR)	Symptomresolution‡,median(ICR)	Stronglyagree oragree	Not	Stronglyagree oragree	Not	Antibioticprescribing,% (n)
Antwerp (Belgium)	164/18/26	26.2 (43)	13.4 (22)	3.7 (6)	93.3 (153)	1.8 (3)	31 (23.8–40.5)	14 (8.5–21)	14.0 (23)	86.0 (141)	93.9 (154)	6.1 (10)	26.8 (44)
Helsinki (Finland)	90/2/25	47.8 (43)	37.8 (34)	11.1 (10)	88.9 (80)	11.1 (10)	32.1 (23.8–44.0)	12.5 (9–19)	36.7 (33)	63.3 (57)	90.0 (81)	10.0 (9)	43.3 (39)
Rotenberg (Germany	181/16/17	25.4 (46)	22.7 (41)	8.8 (16)	90.6 (164)	6.1 (11)	33.3 (23.8–40.5)	18 (11–29)	19.9 (36)	80.1 (145)	100.0 (181)	0.0 (0)	33.7 (61)
Balatonfüred(Hungary)	320/11/11	50.3 (161)	54.4 (174)	31.9 (102)	96.3 (308)	3.4 (11)	26.2 (16.7–35.7)	7 (6–10)	41.9 (134)	58.1 (186)	98.1 (314)	1.9 (6)	74.7 (239)
Milan (Italy)	153/11/12	53.6 (82)	25.5 (39)	3.9 (6)	94.1 (144)	5.2 (8)	19 (13.1–31.0)	11 (7–19)	34.0 (52)	66.0 (86)	96.1 (147)	3.9 (6)	79.1 (121)
Tromso (Norway)	148/11/38	33.1 (49)	27.7 (41)	6.1 (9)	NA	NA	33.3 (26.2–42.9)	13 (8–20)	8.1 (12)	91.9 (136)	83.8 (124)	16.2 (24)	30.4 (45)
Lodz (Poland)	221/9/21	44.3 (98)	47.1 (104)	9.0 (20)	92.8 (205)	7.2 (16)	35.7 (26.2–45.2)	11 (7–16)	41.6 (92)	58.4 (129	86.9 (192)	13.1 (29)	72.4 (160)
Bratislava (Slovakia)	299/5/23	73.2 (219)	65.6 (196)	8.7 (26)	95.0 (284)	2.3 (7)	27.4 (19.0–35.7)	11 (8–16)	49.8 (149)	50.2 (150)	89.6 (268)	10.4 (31)	87.6 (262)
Barcelona (Spain)	169/3/25	28.4 (48)	16.6 (28)	1.2 (2)	95.3 (161)	4.7 (8)	19 (11.9–27.4)	9.5 (6–16.5)	15.4 (26)	84.6 (143)	92.9 (157)	7.1 (12)	18.3 (31)
Mataró (Spain)	179/3/21	33.5 (60)	21.8 (39)	3.4 (6)	96.6 (173)	3.4 (6)	19 (13.1–28.6)	8 (7–15)	15.1 (27)	84.9 (152)	85.5 (153)	14.5 (26)	34.6 (62)
Jönköping (Sweden)	222/12/71	39.6 (88)	45.0 (100)	8.6 (19)	90.1 (200)	9.5 (21)	38.1 (28.6–47.6)	14 (9–24)	22.1 (49)	77.5 (172)	90.5 (201)	9.0 (20)	37.4 (83)
Utrecht (TheNetherlands)	195/11/33	46.2 (90)	40.5 (79)	9.7 (19)	82.6 (161)	6.2 (12)	31 (23.8–42.9)	15 (10–24)	27.1 (53)	72.8 (142)	95.4 (186)	4.6 (9)	42.1 (82)
Cardiff (UnitedKingdom)	181/5/22	63.5 (115)	66.3 (120)	13.3 (24)	98.3 (178)	1.7 (3)	35.7 (26.2–45.2)	14 (8–21)	53.0 (96)	47.0 (85)	96.1 (174)	3.9 (7)	71.8 (130)
Southampton (UnitedKingdom)	168/6/23	42.3 (71)	45.2 (76)	6.0 (10)	85.1 (143)	4.2 (7)	35.7 (26.2–42.9)	14 (9–20)	34.5 (58)	63.1 (106)	84.5 (142)	13.1 (22)	62.5 (105)
Total	2690/123/368	45.1 (1213)	40.6 (1093)	10.2 (275)	92.6 (2354)	4.8 (123)	28.6 (19.0–40.5)	12 (7–19)	31.3 (840)	68.7 (1845)	92.1 (2474)	7.9 (211)	54.4 (1464)

† Score scaled to range between 0 and 100.

‡ Time for patients’ symptom severity scores to drop to 0 in days.

ICR: Interquartile range.

NA: Not applicable.

**Figure 1:**
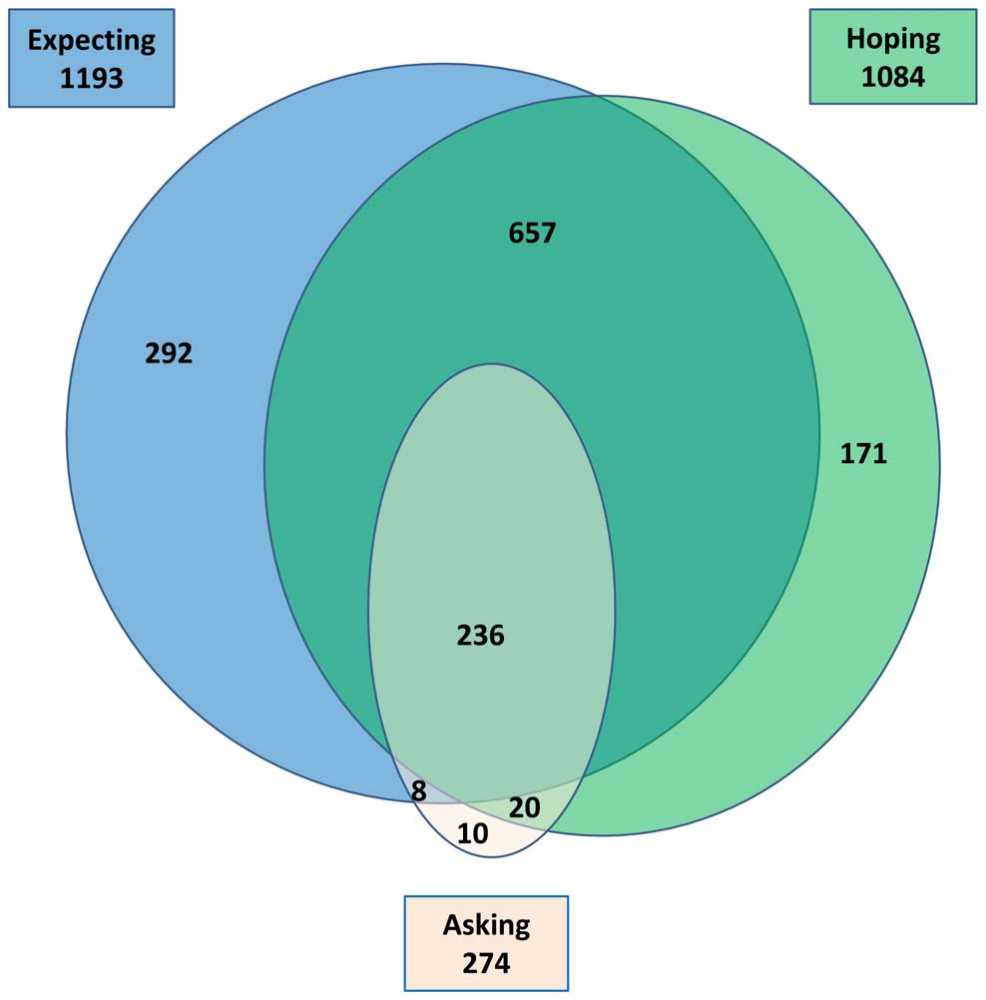
Venn diagram of expectations, hopes and requests for antibiotics in adult outpatients with acute cough.

Overall, 45.1% (1,213) reported that they had been expecting that their clinician would prescribe antibiotics, 40.6% (1,093) reported they had been hoping for antibiotics, and 10.2% (275) reported that they had asked for antibiotics in the index consultation (Tables 1 and S4, Figure 1). Of those expecting antibiotics 25% were not hoping for antibiotics. Of those hoping for antibiotics 18% were not expecting them. And, 74% of those expecting and hoping for were not asking for antibiotics. Regarding clinicians’ perceptions of patient views, 31.3% (840) agreed or strongly agreed with the statement that the patient wanted them to prescribe antibiotics.

92.6% (2,354) reported they were satisfied or very satisfied with their consultation. Clinicians agreed or strongly agreed with the statement that the patient was satisfied with the consultation in 92.1% (2,474).

### Symptom Severity and Resolution

**Figure 2:**
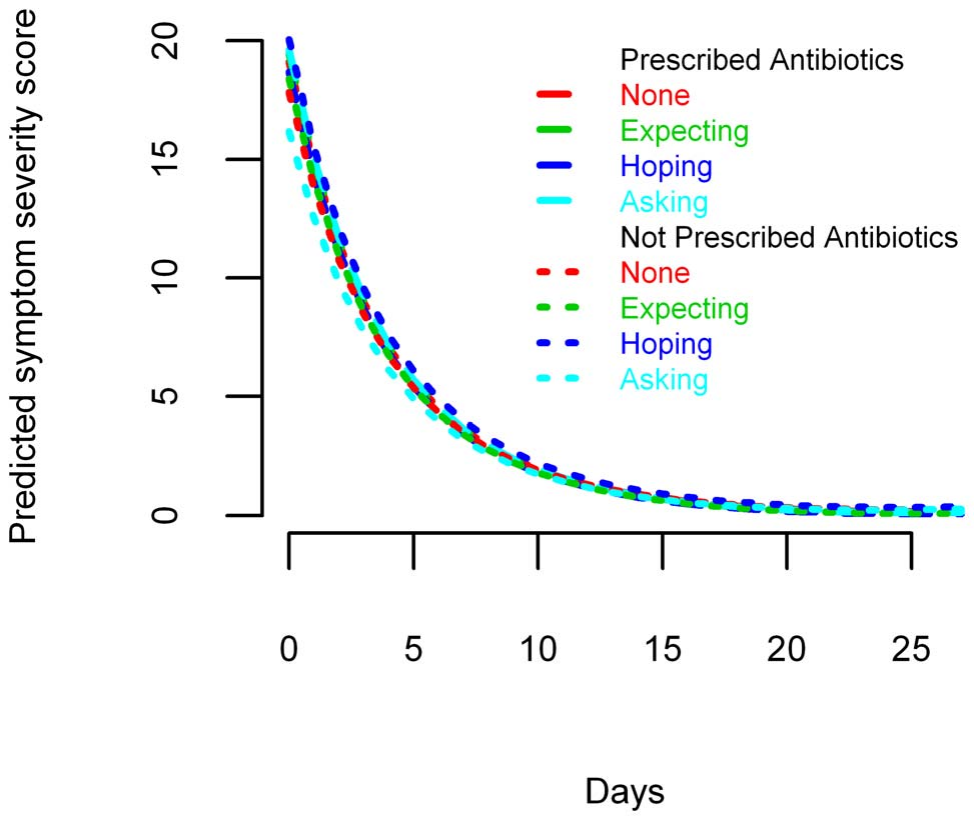
Predicted symptom severity scores over 28 days after presentation for adult outpatients with acute cough.

Symptom severity at baseline and symptom resolution over time were similar for patients expecting, hoping for or asking for antibiotics and those who did not, and these associations were not modified by antibiotic treatment (Table S5). The differences in predicted symptom scores on day 0 and day 7 were small, and not statistically significant between those who were and were not prescribed antibiotics (Figure 2 and Table S6). The predicted symptom resolution for each subgroup is illustrated in Figures 1 and S1.

### Clinicians’ Perception

In this analysis 1211 (46%) patients expected, 1091 (42%) hoped for, and 274 (11%) asked for antibiotics, but clinicians perceived that 31% patients wanted them to prescribe antibiotics (Table 2). The observed agreement was poor, taking into account chance agreement (Kappa = 0.29, 0.32, and 0.21, respectively; Table 3 and Table S7). Otherwise positive agreement was substantially lower than negative agreement.

**Table 2:** Views and satisfaction of adult outpatients with acute cough split by the clinician’s related perceptions question.

		Clinician’s perception				
		This patient wants me to prescribe antibiotics				
Patient		Agree	Do not agree	Cohen’s Kappa	Positive agreement	Negative agreement
**Expecting**	**Yes**	565	646	0.29	0.56	0.72
**Antibiotics** *	**No**	253	1150			
**Hoping for**	**Yes**	538	553	0.32	0.56	0.75
**antibiotics** *	**No**	276	1229			
**Asking**	**Yes**	181	93	0.21	0.33	0.82
**antibiotics** *	**No**	635	1697			
		**This patient was satisfied with the consultation**				
**Patient**		**Agree**	**Do not agree**	**Cohen’s Kappa**	**Positive agreement**	**Negative agreement**
**Satisfied with**	**Yes**	2181	168	0.04	0.94	0.10
**consultation** *	**No**	108	15			

* p<0.0001, McNemar Test.

**Table 3:** Association between patient expectations, hopes and asking for antibiotics and clinician perceptions, and antibiotic prescribing in adult outpatients with acute cough.

Patient views	Odds Ratio (95% CI) for antibiotic prescribing
Expecting antibiotics	2.08 (1.48–2.93)†
Hoping for antibiotics	2.48 (1.73–3.55)†
Asking for antibiotics	0.61 (0.36–1.04)
**Clinician’s perception of**	
Patient wanting to be prescribed antibiotics	
– agree/strongly agree	12.18 (8.31–17.84)†
– neither disagree nor agree/disagree/strongly disagree	Reference category

† p<0.05.

### Antibiotic Prescribing

Patients expecting antibiotics as well as those hoping for them were prescribed antibiotics more frequently than those who were not (OR: 2.08, 95% CI: [1.48, 2.93] and OR: 2.48, 95% CI: [1.73, 3.55], respectively), whereas there was no statistically significant association for patients asking for antibiotics (OR: 0.61, 95% CI: [0.36, 1.04]) (Table 3). Clinician perception significantly influenced antibiotic prescribing (OR: 12.18, 95% CI: [8.31, 17.84]).

### Patient Satisfaction

In this analysis, 2349 (95%) of patients were satisfied with the consultation, and clinicians’ perception was that 93% were satisfied (Table 2). Clinician’s perception of patient satisfaction agreed with patient views for 89% of cases. Taking into account chance agreement however, the observed agreement was very poor (Kappa = 0.04; Table 2 and Table S7). Otherwise positive agreement was very high and negative agreement very low.

No difference in patient satisfaction was found between patients expecting, asking for or prescribed antibiotics and those not. Only patients hoping for antibiotics were less satisfied, when not prescribed an antibiotic (OR : 0.39, 95% CI [0.17, 0.90]. When prescribed antibiotics, patients hoping for antibiotics were more likely to be satisfied (OR interaction term: 3.74, 95% CI [1.16, 12.07]; Table 4). Clinicians’ perception of whether their patient was satisfied with the consultation was not found to be a useful predictor of patients’ self-reported satisfaction regardless of whether they were prescribed an antibiotic or not.

**Table 4:** Association between antibiotic prescribing and patient satisfaction in adult outpatients with acute cough.

Patient views	Odds Ratio (95% CI) for patient satisfaction
Expecting antibiotics	0.51 (0.23–1.13)
Hoping for antibiotics	0.39 (0.17–0.90)†
Asking for antibiotics	2.05 (0.45–9.27)
**Antibiotic prescribing**	0.28 (0.06–1.30)
**Clinician’s perception of**	
Patient satisfaction	
– agree/strongly agree	0.99 (0.33–2.93)
– neither disagree nor agree/disagree/strongly disagree	Reference category
**Significant interaction terms**	
Hoping for antibiotics * Antibiotic prescribing	3.74 (1.16–12.07)†

† p<0.05.

## Discussion

### Summary of Main Findings

In this multi-country study patient views (expectations, hopes or asking for antibiotics) were not associated with symptom severity at presentation, or symptom resolution during the subsequent 28 days, regardless of whether antibiotics were prescribed or not. This implies that these views are not useful for identifying those who will benefit from antibiotic treatment for acute cough. Agreement between these patient views and clinician perceptions of these views was limited, especially positive agreement. Despite this, and controlling for illness severity, patient views and especially clinician perceptions of patient views significantly influenced the antibiotic prescribing decision. Patients were generally satisfied with the consultation. Only those hoping for but not prescribed antibiotics were less satisfied.

### Strengths and Weaknesses

Data collection was part of the first observational GRACE study on the presentation, management and outcome of adults presenting in primary care with LRTI [3]. For this study, we chose to include patients with acute cough, since almost all patients with LRTI have a cough and diagnostic labels such as acute bronchitis are used inconsistently in general practice [32]. The additional eligibility criterion of clinical presentation suggestive of LRTI was added to make those with infection but no cough also eligible. These broad inclusion criteria captured a wide range of patients with LRTI, increasing the generalizability of our results. It has been shown before that there is only limited agreement between the classification of patients with acute cough when applying the criteria defined by Hopstaken et al. and those by Holmes and Macfarlane to define patients with a LRTI, and that the influence of clinician perception that patients wanted antibiotics was similar in acute cough patients with or without an LRTI [7].

The participating clinicians were all part of a primary care research network and so may not have been representative of all primary care clinicians in their country. Similar arguments could be made for their patients. Any selection bias, however, would mean that we might have underestimated the appropriateness, perception and influence of patient views.

As shown in Figure 1, patient expectations, hopes and asking for antibiotics are clearly related, but distinct concepts. None were useful (neither alone or in combination) in identifying those who will benefit from antibiotic treatment for acute cough nor those with complicated recovery. This does not mean that patients’ assessments of the severity of their illness are useless for predicting recovery, but rather that patients are unable to translate this into helpful expectations, hopes or asking for antibiotics. Nevertheless, patient expectations and hopes were independently associated with higher prescribing rates and asking was not.

Patients were given a symptom diary at the time of consulting to be completed afterwards including questions about whether they had expected or hoped for antibiotics before they consulted. It would have been preferable to ask them this before they consulted, but study logistics did not allow this as recruitment generally happened in consultations. This retrospective assessment of expectations and hopes may have been influenced by actual experiences in the consultations. The timing was appropriate for the assessment of patient satisfaction. Because we wanted to assess agreement between patient satisfaction and clinician perception of patient satisfaction, we distinguished between satisfied or very satisfied patients and others. Assessing the influence of patient views and antibiotic prescribing on patient satisfaction, a sensitivity analysis distinguishing between very satisfied patients and all other patients, including satisfied patients, does not alter our conclusions (Table S8).

For our description of agreement between patient views and clinician perceptions, we complemented the Kappa values with measures of positive agreement and negative agreement. Since we were primarily interested in variation in the sample and the suitability of clinicians’ perceptions of patient views in this setting, using Cohen’s Kappa was entirely appropriate, i.e. a reliability measure, while the absolute measures of specific agreement are of interest to clinicians making decisions for individual patients. The latter have not found broad application, despite being extremely helpful for clinicians [33].

Patient diary completion rates ranged from 60% to almost 100% between networks, with a high overall response rate of 80%. It is possible that non-responders deteriorated more than responders, but given the generally benign natural clinical course of this condition, this is unlikely. Since the study involved 13 European countries, there is no guarantee that perceptions of health and symptom reporting were consistent. A study of guideline developers in four European countries found that clinical guidelines reflected differences based on cultural factors such as perceptions of patient expectations [34].

We do not know how cultural differences influenced our results, but a parallel qualitative study showed that in some countries systems are in place for reducing patient expectations [35].

Response bias was not relevant to clinician-recorded data, as there was a 99% completion rate. Clinicians only recorded information, e.g. of the physical examination, that was collected as part of their normal routine. This allowed us to estimate the effect of clinician perceptions of patients wanting antibiotics, controlled only for the presence of the other information that is routinely available. Not requiring additional non-routine investigations also makes this observational study more applicable to everyday practice.

We were interested in the clinicians’ antibiotic prescribing behaviour rather than in the patients’ antibiotic consumption. We have previously shown that more than a third of patients do not adhere to their prescribed antibiotic, but that adherence was not influenced by wanting an antibiotic [25].

### Comparison with Existing Literature

Other studies have reported the lack of a correlation between patients wanting antibiotics or thinking them helpful and the duration of their symptoms [9]–[11]. However, these studies did not take illness severity or antibiotic prescribing into account. Consistent with our findings, a number of studies have reported that clinician perception underestimates patient expectations [10], [11], [20]. Dosh et al. however found that clinician perception if anything overestimated patient expectations for antibiotic prescribing (but their study also included acute sinusitis), and that neither patient expectations nor clinicians’ perceptions of expectations were independent predictors of antibiotic prescribing [12]. Little et al. found that clinician perception of medical need was the strongest factor influencing prescribing as well as other behaviour in the consultation, and the major confounder of the estimates of the other pressures influencing behaviour [36]. Given that clinicians rationalise prescribing, the effect of patient pressure and perceived patient pressure might be underestimated by all studies to date. None of the previous studies assessed agreement between patient expectations, hopes and asking, which we have shown to be clearly related, but distinct features, and clinician perception of these views.

Butler et al. found that clinicians often prescribe antibiotics, even when an antibiotic is not indicated, since most clinicians believe antibiotics present minimal risk and they do not want to risk damaging patient clinician relationships by not prescribing antibiotics [37]. A recent systematic review of qualitative studies corroborates these findings [38].

Welschen et al. showed that, after adjusting for age and gender, patient satisfaction with care is associated with congruence between a clinician’s prescribing decision and patient expectations [17]. However, they did not adjust for illness severity.

### Implications for Policy and Practice

The results of this multicentre study provide the clearest evidence to date that patient expectations and hopes for antibiotics, and especially clinician perceptions of these views, are independent predictors of antibiotic prescribing. We also provide clear evidence that patient views are not associated with illness severity, and therefore are unlikely to represent a rational reason for prescribing antibiotics, and that moreover clinicians are not good at correctly assessing patient views on use of antibiotics. This evidence should now be incorporated into clinical guidelines and tools aimed at improving antibiotic prescribing decisions.

Overall, 95% of patients were satisfied with the consultation. However, satisfaction was lower amongst patients who hoped for an antibiotic but did not receive one. Clinicians need to be reassured that satisfaction amongst patients is generally high. We do not have data on the patterns of communication within these consultations, but it is likely that satisfaction could have been increased through enhanced communication, as has been found in previous studies [14], [39]–[42]. Therefore, our findings also lend support to strategies that seek to enhance communication in these consultations.

## Supporting Information

Figure S1 **Predicted symptom severity scores over 28 days after presentation for adult outpatients with acute cough expecting, hoping for or asking for antibiotics and prescribed an antibiotic or not.**
(TIF)

Table S1 **Translations of the patient views question in the different languages.**
(DOCX)

Table S2 **Patient flow throughout the study.**
(DOCX)

Table S3 **Characteristics of adult outpatients with acute cough included and those not included in the study.**
(DOCX)

Table S4 **Characteristics of adult outpatients with acute cough by primary care network (country).** a. Patient views and satisfaction, symptom severity and resolution. b. Clinician perception and antibiotic prescribing.
(DOCX)

Table S5 **Symptom severity at baseline and symptom resolution over time in adult outpatients with acute cough expecting, hoping for or asking for antibiotics and prescribed an antibiotic or not.**
(DOCX)

Table S6 **Predicted symptom scores for days 0 and 7 for subgroups of adult outpatients with acute cough split by whether they were prescribed antibiotics or not.**
(DOCX)

Table S7 **Agreement between expecting, hoping for or asking for antibiotics or not, and their satisfaction with care among adult outpatients with acute cough and their clinicians’ perception.**
(DOCX)

Table S8 **Association between antibiotic prescribing and patient satisfaction in adult outpatients with acute cough.**
(DOCX)
