# Supplementary figures and images for: Are Patient Views about Antibiotics Related to Clinician Perceptions, Management and Outcome? A Multi-Country Study in Outpatients with Acute Cough
Source: PLoS One. 2013 Oct 23;8(10):e76691. doi: 10.1371/journal.pone.0076691 (PMC3806785; doi:10.1371/journal.pone.0076691)

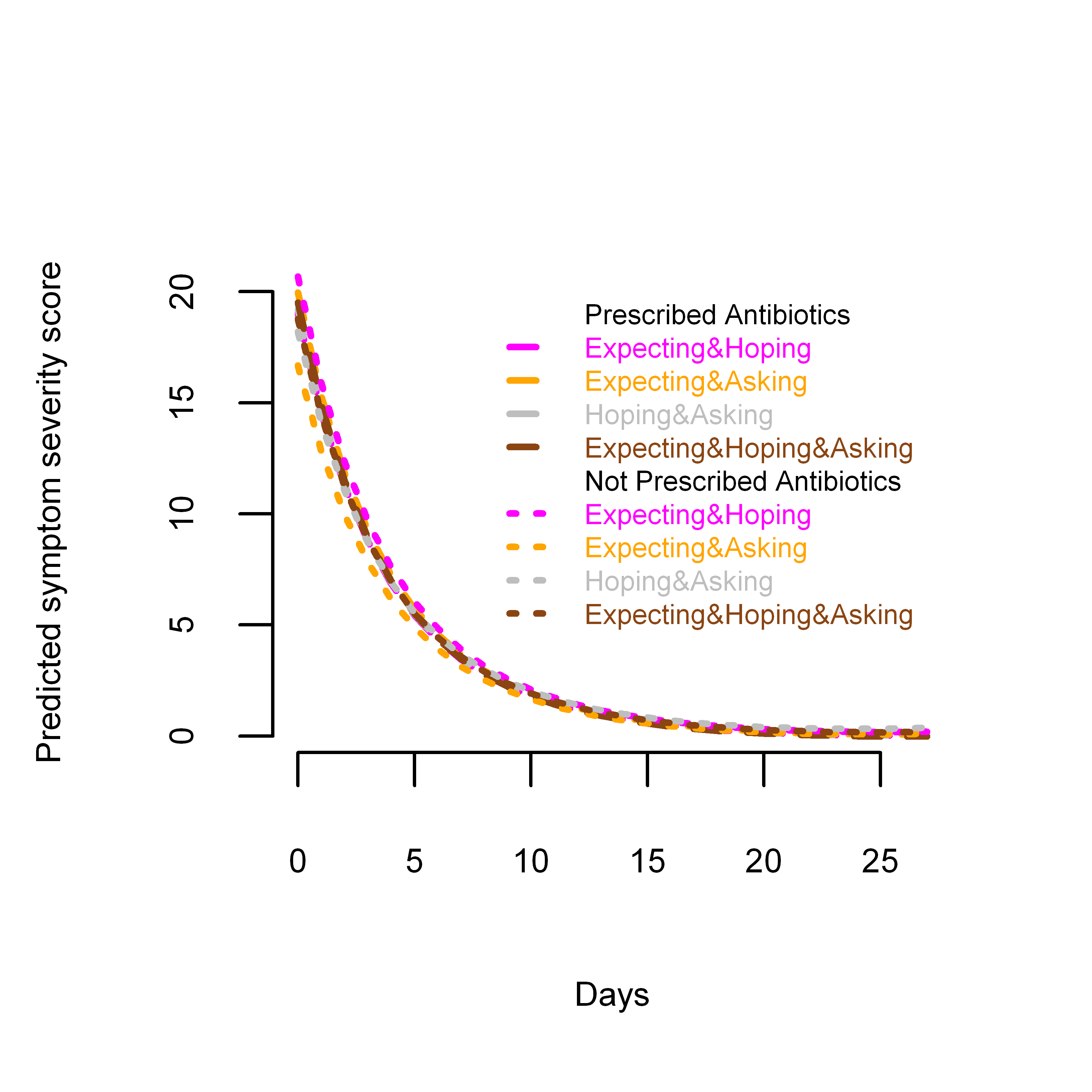

Supplement: Figure S1 — Predicted symptom severity scores over 28 days after presentation for adult outpatients with acute cough expecting, hoping for or asking for antibiotics and prescribed an antibiotic or not. (TIF) [file pone.0076691.s001.tif]
