# Supplementary material for: Are Patient Views about Antibiotics Related to Clinician Perceptions, Management and Outcome? A Multi-Country Study in Outpatients with Acute Cough
Source: PLoS One. 2013 Oct 23;8(10):e76691. doi: 10.1371/journal.pone.0076691 (PMC3806785; doi:10.1371/journal.pone.0076691)
Supplement: Table S1 — Translations of the patient views question in the different languages. (DOCX) [file pone.0076691.s002.docx]

**Table S1. Translations of the patient views question in the different languages.**

| **Language (network)** | **Expecting question** | **Hoping question** | **Asking question** |
| --- | --- | --- | --- |
| English (Cardiff, Southampton) | Were you **expecting** your GP or nurse to  prescribe antibiotics? | Were you **hoping** that your GP or nurse would  prescribe antibiotics? | Did you **ask** your GP or nurse for antibiotics? |
| Dutch (Antwerp, Utrecht) | **Verwachtte** u dat uw huisarts u antibiotica zou voorschrijven? | **Hoopte** u dat uw huisarts u antibiotica zou voorschrijven? | Hebt u uw huisarts **gevraagd** om antibiotica voor te schrijven? |
| Finnish (Helsinki) | **Odotitteko** lääkärin määräävän teille  antibioottia? | **Toivoitteko***,* että lääkäri määräisi Teille  antibioottia? | **Pyysittekö** lääkäriltä antibioottia? |
| German (Rotenberg) | Haben Sie von Ihrem Hausarzt **erwartet**,  dass er Ihnen Antibiotika verschreibt? | Haben Sie **gehofft**, dass Ihr Hausarzt Ihnen  Antibiotika verschreibt? | Haben Sie Ihren Hausarzt nach Antibiotika **gefragt**? |
| Hungarian (Balatonfured) | **Számított arra**, hogy háziorvosa  antibiotikumot ír fel? | **Remélte**, hogy a háziorvosa antibiotikumot ír fel? | Úgy gondolta **kér** antibiotikumot a háziorvostól /  novértol? |
| Italian (Milan) | Vi **aspettavate** che il medico curante prescrivesse  degli antibiotici? | **Speravate** che il medico curante prescrivesse  degli antibiotici? | Avete **chiesto** al medico di prescrivervi antibiotici? |
| Norwegian (Tromso) | **Ventet** du at legen skulle skrive ut  antibiotika? | **Håpet** du at legen skulle skrive ut antibiotika? | **Ba** du legen om å skrive ut antibiotika? |
| Polish (Lodz) | Czy **oczekiwal**/a Pan/i od swojego lekarza  rodzinnego przepisania antybiotyku? | Czy **mial**/a Pan/i nadzieje, ze Pana/Pani lekarz rodzinny przepisze antybiotyk? | Czy **prosil**/a Pan/i swojego lekarza rodzinnego lo antybiotyk? |
| Slovakian (Bratislava) | **Predpokladali ste**, že Vám Váš praktický lekár predpíše antibiotiká? | **Dúfali ste**, že Vám Váš praktický lekár predpíše antibiotiká? | **Požiadali ste** Vášho praktického lekára o antibiotiká? |
| Spanish (Barcelona, Mataro) | **Esperaba** que su médico le recetara  antibióticos? | **Deseaba** que su médico le recetara antibióticos? | **Pidió Usted** que su médico le recetara antibióticos? |
| Swedish (Jonkoping) | **Förväntade** du dig att läkaren skulle skriva  ut antibiotika? | **Hoppades** du att läkaren skulle skriva ut antibiotika? | **Bad** du läkaren om antibiotika? |
