# Supplementary material for: Are Patient Views about Antibiotics Related to Clinician Perceptions, Management and Outcome? A Multi-Country Study in Outpatients with Acute Cough
Source: PLoS One. 2013 Oct 23;8(10):e76691. doi: 10.1371/journal.pone.0076691 (PMC3806785; doi:10.1371/journal.pone.0076691)
Supplement: Table S2 — Patient flow throughout the study. (DOCX) [file pone.0076691.s003.docx]

**Table S2. Patient flow throughout the study.**

**Patients recruited n = 3402**

**Eligible patients n = 3398**

**Returned diary n = 2714**

**Returned CRF n = 3368**

**Returned both forms n =2690**

**Patient satisfaction analysis n = 2287**

**Influence on antibiotic prescribing analysis n = 2380**

**Clinician perception analysis n = 2614**

**Symptom severity and resolution analysis n = 2416**
