# Supplementary material for: Are Patient Views about Antibiotics Related to Clinician Perceptions, Management and Outcome? A Multi-Country Study in Outpatients with Acute Cough
Source: PLoS One. 2013 Oct 23;8(10):e76691. doi: 10.1371/journal.pone.0076691 (PMC3806785; doi:10.1371/journal.pone.0076691)
Supplement: Table S3 — Characteristics of adult outpatients with acute cough included and those not included in the study. (DOCX) [file pone.0076691.s004.docx]

**Table S3. Characteristics of adult outpatients with acute cough included and those not included in the study.***

|  | **Included patients** | **Not-included patients** |
| --- | --- | --- |
| **Male, % (n)** | 36 (973) | 38 (255) |
| **Age, median (IQR)** | 48 (35, 60) | 36 (27, 48)‡ |
| **Temperature, median (IQR)** | 36.8 (36.4, 37.2) | 36.7 (36.2, 37.1)† |
| **Total clinician recorded symptom severity score, median (IQR)** | 26 (22, 31) | 27 (23, 32)† |
| **Antibiotic prescription, % (n)** | 54 (1464) | 46 (312)‡ |
| **Expectations, % (n)** |  |  |
| **- Strongly agree** | 11 (288) | 8 (56) |
| **- Agree** | 21 (552) | 22 (152) |
| **- Neither agree nor disagree** | 29 (770) | 28 (187) |
| **- Disagree** | 24 (649) | 23 (155) |
| **- Strongly disagree** | 16 (426) | 19 (127) |
| **Respiratory comorbidity, % (n)** | 15 (395) | 18 (120) |
| **Diabetes comorbidity, % (n)** | 5 (126) | 2 (12) |
| **Cardiovascular comorbidity, % (n)** | 9 (240) | 4 (26) |
| **Total, n** | 2690 | 678 |

* Patient were included in the analysis if both a case report form and a diary were returned (see Table S2). Proportions were compared using chi-square tests, means using t-tests and where IQRs are reported Wilcoxon rank sum tests used

‡ p<0.001

†p<0.05
