# Supplementary material for: Are Patient Views about Antibiotics Related to Clinician Perceptions, Management and Outcome? A Multi-Country Study in Outpatients with Acute Cough
Source: PLoS One. 2013 Oct 23;8(10):e76691. doi: 10.1371/journal.pone.0076691 (PMC3806785; doi:10.1371/journal.pone.0076691)
Supplement: Table S4 — Characteristics of adult outpatients with acute cough by primary care network (country). a. Patient views and satisfaction, symptom severity and resolution. b. Clinician perception and antibiotic prescribing. (DOCX) [file pone.0076691.s005.docx]

**Table S4. Characteristics of adult outpatients with acute cough by primary care network (country).**

**a. Patient views and satisfaction, symptom severity and resolution .**

|  |  | **Patients** | | | | | | | | | |
| --- | --- | --- | --- | --- | --- | --- | --- | --- | --- | --- | --- |
|  |  |  |  |  | **Satisfied with consultation, % (n)** | | | | |  |  |
|  | **Patients/practises/clinicians, n/n/n** | **Expecting antibiotics, % (n)** | **Hoping for antibiotics, % (n)** | **Asking for antibiotics, % (n)** | **Very satisfied** | **Satisfied** | **Neither satisfied nor dissatisfied** | **Dissatisfied** | **Very dissatisfied** | **Symptom severity score†, median (ICR)** | **Symptom resolution ‡, median (ICR)** |
| Antwerp (Belgium) | 164/18/26 | 26.2 (43) | 13.4 (22) | 3.7 (6) | 59.1 (97) | 34.1 (56) | 1.8 (3) | 0.0 (0) | 0.0 (0) | 31 (23.8-40.5) | 14 (8.5-21) |
| Helsinki (Finland) | 90/2/25 | 47.8 (43) | 37.8 (34) | 11.1 (10) | 40.0 (36) | 48.9 (44) | 8.9 (8) | 1.1 (1) | 1.1 (1) | 32.1 (23.8-44.0) | 12.5 (9-19) |
| Rotenberg (Germany) | 181/16/17 | 25.4 (46) | 22.7 (41) | 8.8 (16) | 49.7 (90) | 40.9 (74) | 3.9 (7) | 1.1 (2) | 1.1 (2) | 33.3 (23.8-40.5) | 18 (11-29) |
| Balatonfüred (Hungary) | 320/11/11 | 50.3 (161) | 54.4 (174) | 31.9 (102) | 56.9 (182) | 39.4 (126) | 2.8 (9) | 0.3 (1) | 0.3 (1) | 26.2 (16.7-35.7) | 7 (6-10) |
| Milan (Italy) | 153/11/12 | 53.6 (82) | 25.5 (39) | 3.9 (6) | 55.6 (85) | 38.6 (59) | 3.9 (6) | 1.3 (2) | 0.0 (0) | 19 (13.1-31.0) | 11 (7-19) |
| Tromso (Norway) | 148/11/38 | 33.1 (49) | 27.7 (41) | 6.1 (9) | NA | NA | NA | NA | NA | 33.3 (26.2-42.9) | 13 (8-20) |
| Lodz (Poland) | 221/9/21 | 44.3 (98) | 47.1 (104) | 9.0 (20) | 45.7 (101) | 47.1 (104) | 6.8 (15) | 0.5 (1) | 0.0 (0) | 35.7 (26.2-45.2) | 11 (7-16) |
| Bratislava (Slovakia) | 299/5/23 | 73.2 (219) | 65.6 (196) | 8.7 (26) | 55.2 (165) | 39,8 (119) | 2.3 (7) | 0.0 (0) | 0.0 (0) | 27.4 (19.0-35.7) | 11 (8-16) |
| Barcelona (Spain) | 169/3/25 | 28.4 (48) | 16.6 (28) | 1.2 (2) | 53.8 (91) | 41.4 (70) | 3.6 (6) | 0.6 (1) | 0.6 (1) | 19 (11.9-27.4) | 9.5 (6-16.5) |
| Mataró (Spain) | 179/3/21 | 33.5 (60) | 21.8 (39) | 3.4 (6) | 54.2 (97) | 42.5 (76) | 3.4 (6) | 0.0 (0) | 0.0 (0) | 19 (13.1-28.6) | 8 (7-15) |
| Jönköping (Sweden) | 222/12/71 | 39.6 (88) | 45.0 (100) | 8.6 (19) | 52.3 (116) | 37.8 (84) | 9.0 (20) | 0.5 (1) | 0.0 (0) | 38.1 (28.6-47.6) | 14 (9-24) |
| Utrecht (The Netherlands) | 195/11/33 | 46.2 (90) | 40.5 (79) | 9.7 (19) | 42.6 (83) | 40.0 (78) | 5.6 (11) | 0.5 (1) | 0.0 (0) | 31 (23.8-42.9) | 15 (10-24) |
| Cardiff (United Kingdom) | 181/5/22 | 63.5 (115) | 66.3 (120) | 13.3 (24) | 71.3 (129) | 27.1 (49) | 1.7 (3) | 0.0 (0) | 0.0 (0) | 35.7 (26.2-45.2) | 14 (8-21) |
| Southampton (United Kingdom) | 168/6/23 | 42.3 (71) | 45.2 (76) | 6.0 (10) | 61.3 (103) | 23.8 (40) | 3.0 (5) | 0.6 (1) | 0.6 (1) | 35.7 (26.2-42.9) | 14 (9-20) |
| **Total** | 2690/123/368 | 45.1 (1213) | 40.6 (1093) | 10.2 (275) | 51.2 (1375) | 36.4 (979) | 4.1 (106) | 0.4 (11) | 0.2 (6) | 28.6 (19.0-40.5) | 12 (7-19) |

**b. Clinician perception and antibiotic prescribing .**

|  | **Clinician perceptions of patient** | | | | | | | | | |  |
| --- | --- | --- | --- | --- | --- | --- | --- | --- | --- | --- | --- |
|  | **Wanting antibiotics, % (n)** | | | | | **Satisfaction, % (n)** | | | | |  |
|  | **Strongly agree** | **Agree** | **Neither agree nor disagree** | **Disagree** | **Strongly disagree** | **Strongly agree** | **Agree** | **Neither agree nor disagree** | **Disagree** | **Strongly disagree** | **Antibiotic prescribing, % (n)** |
| Antwerp (Belgium) | 5.5 (9) | 8.5 (14) | 17.7 (29) | 28.7 (47) | 39.6 (65) | 22.6 (37) | 71.3 (117) | 6.1 (10) | 0.0 (0) | 0.0 (0) | 26.8 (44) |
| Helsinki (Finland) | 11.1 (10) | 25.6 (23) | 10.0 (9) | 23.3 (21) | 30.0 (27) | 37.8 (34) | 52.2 (47) | 7.8 (7) | 2.2 (2) | 0.0 (0) | 43.3 (39) |
| Rotenberg (Germany) | 3.3 (6) | 16.6 (30) | 18.2 (33) | 50.3 (91) | 11.6 (21) | 35.4 (64) | 64.6 (117) | 0.0 (0) | 0.0 (0) | 0.0 (0) | 33.7 (61) |
| Balatonfüred (Hungary) | 15.9 (51) | 25.9 (83) | 27.8 (89) | 21.2 (68) | 9.1 (29) | 32.2 (103) | 65.9 (211) | 1.9 (6) | 0.0 (0) | 0.0 (0) | 74.7 (239) |
| Milan (Italy) | 10.5 (16) | 23.5 (36) | 56.2 (86) | 8.5 (13) | 1.3 (2) | 32.7 (50) | 63.4 (97) | 3.9 (6) | 0.0 (0) | 0.0 (0) | 79.1 (121) |
| Tromso Norway) | 1.4 (2) | 6.8 (10) | 39.2 (58) | 29.7 (44) | 23.0 (34) | 18.9 (28) | 64.9 (96) | 16.2 (24) | 0.0 (0) | 0.0 (0) | 30.4 (45) |
| Lodz (Poland) | 14.5 (32) | 27.1 (60) | 26.2 (58) | 19.9 (44) | 12.2 (27) | 16.7 (37) | 70.1 (155) | 12.7 (28) | 0.5 (1) | 0.0 (0) | 72.4 (160) |
| Bratislava (Slovakia) | 16.7 (50) | 33.1 (99) | 26.1 (78) | 17.7 (53) | 6.4 (19) | 23.7 (71) | 65.9 (197) | 10.0 (30) | 0.3 (1) | 0.0 (0) | 87.6 (262) |
| Barcelona (Spain) | 3.6 (6) | 11.8 (20) | 29.0 (49) | 43.2 (73) | 12.4 (21) | 30.2 (51) | 62.7 (106) | 7.1 (12) | 0.0 (0) | 0.0 (0) | 18.3 (31) |
| Mataró (Spain) | 5.0 (9) | 10.1 (18) | 38.5 (69) | 21.2 (38) | 25.1 (45) | 23.5 (42) | 62.0 (111) | 13.4 (24) | 1.1 (2) | 0.0 (0) | 34.6 (62) |
| Jönköping (Sweden) | 11.7 (26) | 10.4 (23) | 22.1 (49) | 19.8 (44) | 35.6 (79) | 23.9 (53) | 66.7 (148) | 8.6 (19) | 0.5 (1) | 0.0 (0) | 37.4 (83) |
| Utrecht (The Netherlands) | 8.7 (17) | 18.5 (36) | 25.1 (49) | 27.2 (53) | 20.5 (40) | 23.6 (46) | 71.8 (140) | 4.6 (9) | 0.0 (0) | 0.0 (0) | 42.1 (82) |
| Cardiff (United Kingdom) | 21.0 (38) | 32.0 (58) | 35.9 (65) | 8.8 (16) | 2.2 (4) | 24.9 (45) | 71.3 (129) | 3.9 (7) | 0.0 (0) | 0.0 (0) | 71.8 (130) |
| Southampton (United Kingdom) | 9.5 (16) | 25.0 (42) | 29.2 (49) | 26.2 (44) | 7.7 (13) | 8.3 (14) | 76.2 (128) | 13.1 (22) | 0.0 (0) | 0.0 (0) | 62.5 (105) |
| **Total** | 10.7 (288) | 20.5 (552) | 28.6 (770) | 24.1 (649) | 15.8 (426) | 25.1 (675) | 66.9 (1799) | 7.6 (204) | 0.3 (7) | 0.0 (0) | 54.4 (1464) |

**†**: Score scaled to range between 0 and 100.

‡: Time for patients’ symptom severity scores to drop to 0 in days.
ICR: Interquartile range.
NA: Not applicable
