# Supplementary material for: Are Patient Views about Antibiotics Related to Clinician Perceptions, Management and Outcome? A Multi-Country Study in Outpatients with Acute Cough
Source: PLoS One. 2013 Oct 23;8(10):e76691. doi: 10.1371/journal.pone.0076691 (PMC3806785; doi:10.1371/journal.pone.0076691)
Supplement: Table S5 — Symptom severity at baseline and symptom resolution over time in adult outpatients with acute cough expecting, hoping for or asking for antibiotics and prescribed an antibiotic or not. (DOCX) [file pone.0076691.s006.docx]

**Table S5. Symptom severity at baseline and symptom resolution over time in adult outpatients with acute cough expecting, hoping for or asking for antibiotics and prescribed an antibiotic or not.**

|  | **Symptom severity at baseline for those not prescribed antibiotics** | **Symptom resolution over 28 days for those not prescribed antibiotics** | **Symptom severity at baseline for those prescribed antibiotics** | **Symptom resolution over 28 days for those prescribed antibiotics** |
| --- | --- | --- | --- | --- |
|  | Coefficient (95%CI)* | Coefficient (95%CI)* | Coefficient (95%CI)* | Coefficient (95%CI)* |
| Expecting antibiotics | 0.04 (-0.12 , 0.19) | -0.01 (-0.01 , 0.00) | -0.02 (-0.21 , 0.17) | 0.00 (-0.01 , 0.01) |
| Hoping for antibiotics | 0.11 (-0.06 , 0.28) | 0.00 (-0.01 , 0.01) | -0.13 (-0.34 , 0.08) | 0.00 (-0.01 , 0.01) |
| Asking for antibiotics | -0.10 (-0.36 , 0.17) | 0.00 (-0.01 , 0.02) | 0.12 (-0.18 , 0.42) | -0.01 (-0.02 , 0.01) |

* The three variables (expecting, hoping for, and asking for antibiotics) were included in a single model. The coefficients were derived from a three-level hierarchical ARMA (1,1) model [28], with logged daily symptom scores nested within patients nested within clinicians, and fitting three-way interaction terms between being prescribed an antibiotic, time measured in days and each of expecting, hoping for or asking for antibiotics. To illustrate the practical implication of these coefficients, predicted symptoms scores for days 0 and 7 based on an average patient are presented in Table S6.

† p<0.05
