# Supplementary material for: Are Patient Views about Antibiotics Related to Clinician Perceptions, Management and Outcome? A Multi-Country Study in Outpatients with Acute Cough
Source: PLoS One. 2013 Oct 23;8(10):e76691. doi: 10.1371/journal.pone.0076691 (PMC3806785; doi:10.1371/journal.pone.0076691)
Supplement: Table S6 — Predicted symptom scores for days 0 and 7 for subgroups of adult outpatients with acute cough split by whether they were prescribed antibiotics or not. (DOCX) [file pone.0076691.s007.docx]

**Table S6. Predicted symptom scores for days 0 and 7 for subgroups of adult outpatients with acute cough split by whether they were prescribed antibiotics or not.**

|  | **Predicted symptom scores** † | | | |
| --- | --- | --- | --- | --- |
|  | **Day 0** | | **Day 7** | |
|  | **Not prescribed antibiotics** | **Prescribed antibiotics** | **Not prescribed antibiotics** | **Prescribed antibiotics** |
| **Not expecting/hoping for/asking for antibiotics** | 17.8 | 19.1 | 3.5 | 3.6 |
|  |  |  |  |  |
| **Expecting antibiotics** | 18.4 | 19.4 | 3.4 | 3.6 |
|  |  |  |  |  |
| **Hoping for antibiotics** | 20.0 | 18.7 | 4.0 | 3.4 |
|  |  |  |  |  |
| **Asking for antibiotics** | 16.2 | 19.6 | 3.2 | 3.7 |
|  |  |  |  |  |
| **Expecting and hoping for antibiotics** | 20.7 | 19.0 | 3.9 | 3.4 |
|  |  |  |  |  |
| **Expecting, hoping for and asking for antibiotics** | 16.7 | 20.0 | 3.1 | 3.7 |
|  |  |  |  |  |
| **Expecting and asking for antibiotics** | 18.2 | 19.2 | 3.6 | 3.5 |
|  |  |  |  |  |
| **Hoping and asking for antibiotics** | 18.8 | 19.5 | 3.6 | 3.5 |

† In order to illustrate the practical implication of the coefficients presented in Table S5, we predicted symptoms scores for days 0 and 7 based on an average patient, i.e. an adult with acute cough, moderately severe phlegm production, feeling moderately unwell and normal temperature (≥36°c and ≤37.2°c), median age (45), median days waited before presentation (5), no comorbidities and non-smoker.
