# Supplementary material for: Are Patient Views about Antibiotics Related to Clinician Perceptions, Management and Outcome? A Multi-Country Study in Outpatients with Acute Cough
Source: PLoS One. 2013 Oct 23;8(10):e76691. doi: 10.1371/journal.pone.0076691 (PMC3806785; doi:10.1371/journal.pone.0076691)
Supplement: Table S7 — Agreement between expecting, hoping for or asking for antibiotics or not, and their satisfaction with care among adult outpatients with acute cough and their clinicians’ perception. (DOCX) [file pone.0076691.s008.docx]

**Table S7. Agreement between expecting, hoping for or asking for antibiotics or not, and their satisfaction with care, among adult outpatients with acute cough, and their clinicians’ perception.**

| **Cohen's Kappa** | **Total** | **Cardiff** | | **Southampton** | **Utrecht** | | **Barcelona** | | **Mataró** | **Rotenburg** | | **Balatonfüred** | | **Antwerp** | **Lodz** | | **Milan** | | **Jonkoping** | **Tromso** | | **Helsinki** | **Bratislava** |  |  |  |  |
| --- | --- | --- | --- | --- | --- | --- | --- | --- | --- | --- | --- | --- | --- | --- | --- | --- | --- | --- | --- | --- | --- | --- | --- | --- | --- | --- | --- |
| **- Expects** | **0.29** | 0.20 | | 0.25 | 0.27 | | 0.19 | | 0.11 | 0.22 | | 0.36 | | 0.22 | 0.34 | | 0.23 | | 0.33 | 0.19 | | 0.33 | 0.05 |  |  |  |  |
| **- Hopes for** | **0.32** | 0.21 | | 0.32 | 0.32 | | 0.16 | | 0.15 | 0.34 | | 0.36 | | 0.42 | 0.31 | | 0.11 | | 0.30 | 0.20 | | 0.52 | 0.00 |  |  |  |  |
| **­- Asks for** | **0.21** | 0.14 | | 0.11 | 0.26 | | 0.05 | | 0.01 | 0.18 | | 0.42 | | 0.09 | 0.16 | | 0.07 | | 0.36 | 0.23 | | 0.31 | * |  |  |  |  |
|  |  | |  | | |  | |  | | |  | |  | | |  | |  | | |  | |  |  |  |  |  |
| **- Satisfied** | **0.04** | * | | * | 0.05 | | * | | 0.07 | 0.00 | | 0.10 | | * | 0.04 | | * | | * | NA | | 0.12 | 0.23 |  |  |  |  |
|  |  | |  | | |  | |  | | |  | |  | | |  | |  | | |  | |  |  |  |  |  |
| **Positive agreement** | | | |  |  | |  | |  |  | |  | |  |  | |  | |  |  | |  |  |  |  |  |  |
| **- Expects** | **0.56** | 0.66 | | 0.56 | 0.52 | | 0.35 | | 0.30 | 0.40 | | 0.65 | | 0.37 | 0.62 | | 0.55 | | 0.52 | 0.30 | | 0.61 | 0.62 |  |  |  |  |
| **- Hopes for** | **0.56** | 0.68 | | 0.61 | 0.55 | | 0.30 | | 0.30 | 0.48 | | 0.66 | | 0.50 | 0.61 | | 0.37 | | 0.51 | 0.30 | | 0.70 | 0.58 |  |  |  |  |
| **- Asks for** | **0.33** | 0.32 | | 0.20 | 0.38 | | 0.07 | | 0.06 | 0.28 | | 0.63 | | 0.14 | 0.29 | | 0.14 | | 0.44 | 0.29 | | 0.43 | 0.14 |  |  |  |  |
|  |  | |  | | |  | |  | | |  | |  | | |  | |  | | |  | |  |  |  |  |  |
| **- Satisfied** | **0.94** | 0.97 | | 0.90 | 0.94 | | 0.94 | | 0.91 | 0.97 | | 0.98 | | 0.96 | 0.90 | | 0.95 | | 0.90 | NA | | 0.91 | 0.95 |  |  |  |  |
|  |  | |  | | |  | |  | | |  | |  | | |  | |  | | |  | |  |  |  |  |  |
| **Negative agreement** | | | |  |  | |  | |  |  | |  | |  |  | |  | |  |  | |  |  |  |  |  |  |
| **- Expects** | **0.72** | 0.53 | | 0.68 | 0.69 | | 0.82 | | 0.77 | 0.82 | | 0.70 | | 0.83 | 0.71 | | 0.64 | | 0.79 | 0.81 | | 0.71 | 0.36 |  |  |  |  |
| **- Hopes for** | **0.75** | 0.52 | | 0.70 | 0.74 | | 0.86 | | 0.84 | 0.86 | | 0.69 | | 0.92 | 0.69 | | 0.72 | | 0.75 | 0.84 | | 0.82 | 0.39 |  |  |  |  |
| **- Asks for** | **0.82** | 0.66 | | 0.79 | 0.86 | | 0.92 | | 0.90 | 0.88 | | 0.78 | | 0.91 | 0.76 | | 0.79 | | 0.90 | 0.94 | | 0.82 | 0.64 |  |  |  |  |
|  |  | |  | | |  | |  | | |  | |  | | |  | |  | | |  | |  |  |  |  |  |
| **- Satisfied** | **0.10** | 0.00 | | 0.00 | 0.10 | | 0.00 | | 0.13 | 0.00 | | 0.12 | | 0.00 | 0.13 | | 0.00 | | 0.05 | NA | | 0.21 | 0.26 |  |  |  |  |

* Kappa is not calculated for this dataset because observed concordance is smaller than mean-chance concordance.

NA: Not applicable
