# Supplementary material for: Are Patient Views about Antibiotics Related to Clinician Perceptions, Management and Outcome? A Multi-Country Study in Outpatients with Acute Cough
Source: PLoS One. 2013 Oct 23;8(10):e76691. doi: 10.1371/journal.pone.0076691 (PMC3806785; doi:10.1371/journal.pone.0076691)
Supplement: Table S8 — Association between antibiotic prescribing and patient satisfaction in adult outpatients with acute cough. (DOCX) [file pone.0076691.s009.docx]

**Table S8. Association between antibiotic prescribing and patient satisfaction in adult outpatients with acute cough.**

| **Patient views** | | **Odds Ratio (95% CI) for patient satisfaction** | | | **Odds Ratio  (95% CI) for patient satisfaction** |
| --- | --- | --- | --- | --- | --- |
|  | **(satisfied and very satisfied vs other)** | | | | **(very satisfied vs other)** |
| Expecting antibiotics | | 0.51 (0.23 – 1.13) | | | 0.70 (0.47 – 1.04) |
| Hoping for antibiotics | | 0.39 (0.17 – 0.90)† | | | 0.62 (0.40 – 0.97) † |
| Asking for antibiotics | | 2.05 (0.45 – 9.27) | | | 2.13 (1.05 – 4.32) † |
|  | |  | | |  |
| **Antibiotic prescribing** | | 0.28 (0.06 – 1.30) | | | 0.88 (0.42 – 1.85 ) |
|  | |  | | |  |
| **Clinician’s perception of** | |  | | |  |
| Patient satisfaction | | |  | |  |
| – agree/strongly agree | | | | 0.99 (0.33 – 2.93) | 1.18 (0.72 – 1.93) |
| – neither disagree nor agree/ disagree/strongly disagree | | | | Reference category | Reference category |
|  | | | |  |  |
| **Significant interaction terms** | | | |  |  |
| Hoping for antibiotics Antibiotic prescribing | | | | 3.74 (1.16 - 12.07)† | 1.75 (1.02 -2.99) † |
|  | | | |  |  |

† p<0.05
